# Isorhamnetin alleviates symptoms and inhibits oxidative stress levels in rats with pulmonary arterial hypertension

**DOI:** 10.22038/ijbms.2024.75860.16421

**Published:** 2024

**Authors:** Yefeng Chen, Ping Ma, Lei Bo, Yingjie Lv, Wei Zhou, Ru Zhou

**Affiliations:** 1 School of Clinical Medicine, Ningxia Medical University,Yinchuan, China; 2 General Hospital of Ningxia Medical University, Yinchuan, China; 3 Department of Foreign Language Teaching, Ningxia Medical University, Yinchuan, China; 4 School of Public Health and Management, Ningxia Medical University, Yinchuan, China; 5 People’s Hospital of Ningxia Hui Autonomous Region, Yinchuan, China; 6 Department of Pharmacology, College of Pharmacy, Ningxia Medical University, Yinchuan, China; 7 Key Laboratory of Hui Ethnic Medicine Modernization, Ministry of Education, Ningxia Medical University, Yinchuan, China; 8 NHC Key Laboratory of Metabolic Cardiovascular Diseases Research, Ningxia Medical University, Yinchuan 750004, China; 9 Ningxia Characteristic Traditional Chinese Medicine Modernization Engineering Technology Research Center, Ningxia Medical University, Yinchuan, China; # These authors contributed equally to this work

**Keywords:** 5-Hydroxytryptamine Isorhamnetin, Machine-processed Oxidative stress, Pulmonary arterial- hypertension, Sea buckthorn

## Abstract

**Objective(s):**

Pulmonary arterial hypertension (PAH) is a malignant pulmonary vascular disease with high mortality. Isorhamnetin (ISO), one of the main natural flavonoids extracted from sea buckthorn, has pharmacological effects such as anti-inflammatory, anti-proliferative and antioxidant. This study aimed to investigate the protective effect of ISO on PAH and its relationship with the phosphorylation of the c-Src tyrosine kinase (p-c-src)/NOX1 signaling pathway.

**Materials and Methods:**

Ninety-five rats were randomly divided into five groups. The normal group received only a subcutaneous injection of saline, while the other groups received a subcutaneous injection of monocrotaline(MCT) (60 mg/kg) to establish a PAH model. The treatment group received ISO (50, 100, 150 mg/kg/d) treatment for 21 days, and after 21 days, all rat lung tissues were separated.

**Results:**

The results showed that ISO could significantly improve the hemodynamics of MCT-induced PAH rats, such as mean pulmonary artery pressure (mPAP) and right ventricular systolic pressure (RVSP), and had inhibitory effects on right ventricular hypertrophy in PAH rats, and on pulmonary vascular remodeling in PAH rats. In addition, ISO can reduce the content of 5-hydroxytryptamine (5-HT) in PAH rats, increase the expression of Nrf2 protein in the lung tissue of PAH rats, activate the antioxidant system, enhance the activity of SOD in lung tissue of PAH rats, and inhibit NOX1, 5-HTT, p-c-src and Proliferating Cell Nuclear Antigen(PCNA) protein expression, and decrease MDA content.

**Conclusion:**

Our research confirmed the therapeutic effect of ISO on MCT-induced PAH rats, which may be related to regulating the p-c-src/NOX1 signaling pathway.

## Introduction

Pulmonary arterial hypertension (PAH) is a malignant pulmonary vascular disease with a low cure rate and high mortality (1). With the proliferation and remodeling of small pulmonary artery wall cells, pulmonary vasoconstriction, pulmonary vascular wall remodeling, and the formation of *in situ* thromboembolism, the pulmonary vascular resistance continues to rise, which in turn leads to right heart failure until the patient’s death (2). More studies have shown that oxidative stress is involved in the formation of PAH. When the body is subjected to various harmful stimuli, the excessive reactive oxygen species (ROS) and other highly active molecules produced in the body exceed the ability of the body to clear oxides, resulting in tissue damage, which is called oxidative stress. ROS can regulate cell signal transduction, especially in reactions where nicotinamide adenine dinucleotide phosphate (NADPH) oxidase is the primary source of reactive oxygen species. This indicates that NADPH oxidase and its downstream components in the ROS signal transduction pathway may be potential targets for treating PAH (3, 4). 5-Hydroxytryptamine (5-HT) is a natural pulmonary vasoconstrictor and smooth muscle cell mitogen, which has been found to be related to the pathogenesis of PAH. 5-HT can induce nicotinamide adenine dinucleotide phosphate oxidase-1 (NOX1) regulated by cellular Src-related kinases, which in turn induces the dysregulation of ROS and Nuclear Factor erythroid 2-Related Factor 2 (Nrf2), leading to increased post-translational oxidative modification of proteins in human pulmonary artery smooth muscle cells and activation of redox-sensitive signaling pathways (5-7). 

 Isorhamnetin (ISO) is a natural flavonoid extracted from the traditional Chinese medicine sea buckthorn widely grown in the Ningxia Hui Autonomous Region. ISO has various biological effects such as anti-oxidation, anti-inflammatory, anti-proliferation, and anti-tumor (8, 9). Studies have confirmed that ISO can relieve the oxidative stress load of cardiomyocytes, inhibit intracellular ROS production and lipid peroxidation, and increase the level of antioxidant enzymes in the cell. ISO increases the antioxidant capacity of cardiomyocytes and inhibits cardiomyocyte apoptosis by activating the Nrf2 pathway (10). Another study proved that ISO exerts a neuroprotective effect on Streptozotocin-induced diabetic rats by reducing oxidative stress (11). In addition, studies have confirmed that ISO can reduce acute lung injury in mice (12). Previous studies have found the protective effect of ISO on PAH rats, but the specific mechanism of ISO on PAH still needs more research (13).

In view of the above studies, this research aims to investigate the therapeutic effect of ISO on PAH and explore whether it has a therapeutic effect on monocrotaline (MCT)-induced PAH through the phosphorylation of the c-Src tyrosine kinase (p-c-src)/NOX1 signaling pathway.

## Materials and Methods

***Animals and reagents***

Male Sprague-Dawley rats (190-210 g) from the Experimental Animal Center were used. The experiment was approved by the Experimental Animal Committee. The rats were housed in a temperature-controlled room with a 12:12 day-night cycle. ISO with a purity of greater than 98.47%, revealed by HPLC analysis, was purchased from Beijing Zhongke Quality Inspection Biological Co., Ltd., and MCT was provided by Sigma Company.

***Experimental procedure***

There were 95 rats randomly divided into five groups: Control group (n=19); Monocrotaline (MCT) group (n=19); ISO (50 mg/kg/d)+MCT groups (n=19); ISO (100 mg/kg/d)+MCT groups (n=19), and ISO (150 mg/kg/d)+MCT groups (n=19). Throughout the entire experimental process, each rat was placed in an environment with a relatively constant temperature without restricting drinking and eating. On the first day of modeling, except for the control group, the other rats were conducted with a single subcutaneous injection of MCT (60 mg/kg) to establish a PAH model. Rats were fed normally from the first day to the 21st day after the completion of the modeling. After the end of modeling, and from 21 d to 42 d, the rats in the treatment group were administered ISO (50,100,150 mg/kg/d) daily. The rats in the control group and the MCT group were given the same dose of normal saline (NS) every day. 

***Hemodynamic measurements***

On the second day after the last administration in rats, rats were anesthetized with a single intraperitoneal injection of 20% urethane (0.2g/100 g) and fixed on the operating table, separating the right external jugular vein of the rat, and inserting a polyethylene catheter into the right external jugular vein to connect to a pressure sensor. MPA cardiac function analysis system (Alcott Biotech, Shanghai, China) was used to record right ventricular systolic pressure (RVSP) and mean pulmonary artery pressure (mPAP).

***Tissue preparation***

After measuring hemodynamics, we removed the lung tissue and weighed it; the left lower lobes were fixed with 4% paraformaldehyde for 48 hr and stained with hematoxylin and eosin (H&E). The right lung was frozen and stored in a -80 ^°^C freezer in preparation for the next experiment.

***Histopathological analysis***

We fixed the removed lung tissue with 4% Paraformaldehyde for 48 hr. When the fixed time was over, the lung tissue was embedded in paraffin and sliced 4 μm. Then, H&E staining was performed on the sections. The stained sections were observed with an optical microscope (Olympus BX-51, Tokyo, Japan). Pulmonary artery remodeling was observed using H&E staining. The following two indicators reflecting pulmonary artery remodeling were measured: (a) pulmonary artery wall thickness (WT%)=100%×(outer diameter−inner diameter)/outer diameter; (b) pulmonary artery wall area (WA%)=100%×(cross-sectional area-lumen area)/tube wall cross-sectional area.

***Right ventricular mass index***

After hemodynamic parameters were detected, the rats were immediately executed for the lungs and heart. The heart‘s right ventricle (RV) was washed in NS and blotted dry with filter paper. The RV‘s weight was weighed, and the right ventricular mass index=RVW/BMW was calculated.

***Measurements of biomarkers of oxidative stress***

The frozen lung tissue was put into a 1.5 ml grinding tube according to the ratio of frozen tissue (NS 1:9). The grinding tubes were put into a fully automatic freeze grinding machine for a 10% lung tissue homogenate according to the parameters of the instructions. After centrifugation at 10000 r/min for 10 min, the supernatant was collected for the determination of Superoxide dismutase (SOD) activity and malonaldehyde (MDA).

***Determination of 5-HT content***

The frozen lung tissue was taken and put into a 1.5 ml grinding tube according to the ratio of frozen tissue (PBS 1:9). The grinding tubes were then put into a fully automatic freezing grinder according to the instructions‘ parameters and then centrifuged at 10000 r/min for 10 min. The supernatant was taken and packaged, and then the 5-HT content was determined according to the operation steps and methods of the Elisa kit.

***Western blot***

Proteins were extracted from lung tissue following the instructions of the BCA protein detection kit (KeyGEN Biotechnology Company, Jiangsu, China). Then, 8% protein lysates were separated by SDS-PAGE gel and western blotted onto PVDF membranes using the BIO-RAD system. The PVDF membrane was blocked with 5% nonfat milk in PBST for two hours at room temperature. Subsequently, rabbit monoclonal anti-NOX1 antibody (1:1000), rabbit polyclonal anti-Nrf2 antibody (1:500), p-c-src antibody (1:1000), 5-hydroxytryptamine transporter (5-HTT) antibody (1:1000), Proliferating Cell Nuclear Antigen(PCNA) antibody (1:1000) was incubated at 4 ^°^C overnight, then incubated with goat anti-rabbit IgG (1:5000) as secondary antibody and protein bands were visualized with ECL luminescence chromogenic solution analysis of protein bands for each factor using QuantityOne software (Bio-Rad, CA, USA).

***Statistical analysis***

Data are presented as mean±standard error of the mean. All tests were carried out with SPSS version 24.0 statistical software. Statistical analyses were performed by One-way ANOVA followed by Fisher‘s least significant difference test (LSD) mean data for multiple comparisons; *P*<0.05 and *P*< 0.01 were considered statistically significant.

## Results

***Effects of*** ***ISO*** ***on hemodynamics in rats***

**Figure 1:**
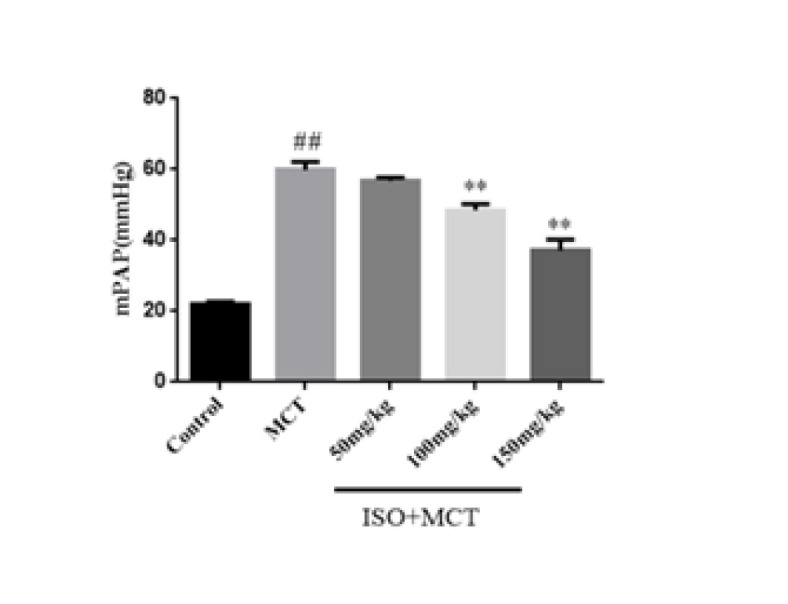
Effect of isorhamnetin on mean pulmonary arterial pressure (mPAP) in male SD rats

**Figure 2:**
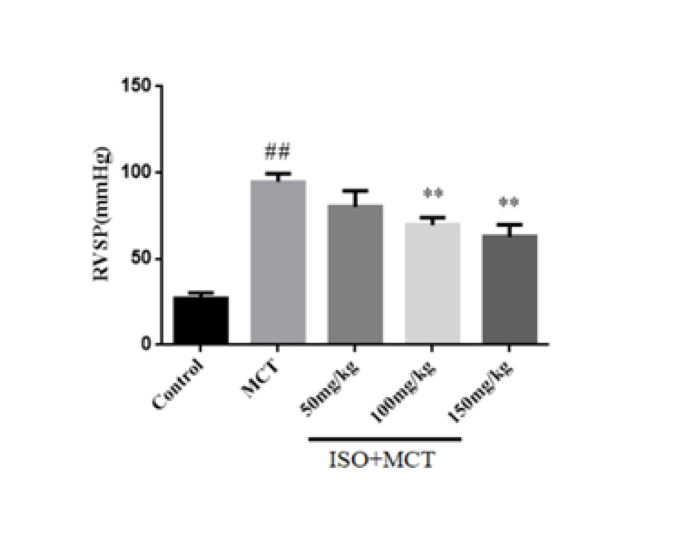
Effect of isorhamnetin on right ventricular systolic pressure (RVSP) in male SD rats

Firstly, a rat pulmonary hypertension model was constructed using MCT, and its success was verified through right heart catheterization. mPAP represents the gold indicator of pulmonary vascular pressure. Previously, the hemodynamics results obtained from the pharmacological experiments of our research group on the protective effect of ISO on PAH were consistent with those of this experiment (13). The results analyzed by the MPA system showed that compared with the control group, mPAP and RVSP were significantly increased in the model group, which indicated that the rat model of PAH was successfully constructed (Figure 1 and Figure 2). Compared with the model group, ISO groups (100, 150 mg/kg) could inhibit the increase of pulmonary arterial pressure, which indicated that ISO could improve hemodynamics (Figures 1 and 2).

***Effects of*** ***ISO*** ***on right ventricular mass index***

**Figure 3:**
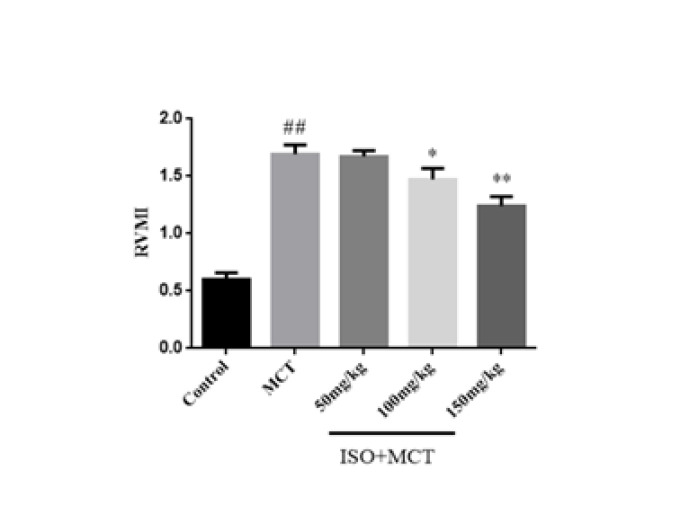
Effect of isorhamnetin on Right ventricular mass index (RVMI) in male SD rats

Previously, the Right ventricular hypertrophy (RVHI) obtained from the pharmacological experiments of our research group on the protective effect of ISO on PAH was consistent with those of this experiment (13). However, this experiment chose a similar indicator, the right ventricular mass index (RVMI). RVMI can express the degree of cardiac hypertrophy, and the degree of changes in the heart of rats after the disease can be seen by observing RVMI. Organ weighing results showed that, compared with the control group, the right ventricular mass index (RVMI) in the model group was significantly increased, indicating that right ventricular hypertrophy occurred in the PAH rats (Figure 3). Compared with the model group, ISO (150 mg/kg) could inhibit the increase of RVMI (Figure 3).

***Effect of*** ***ISO*** ***on pulmonary vascular remodeling***

**Figure 4:**
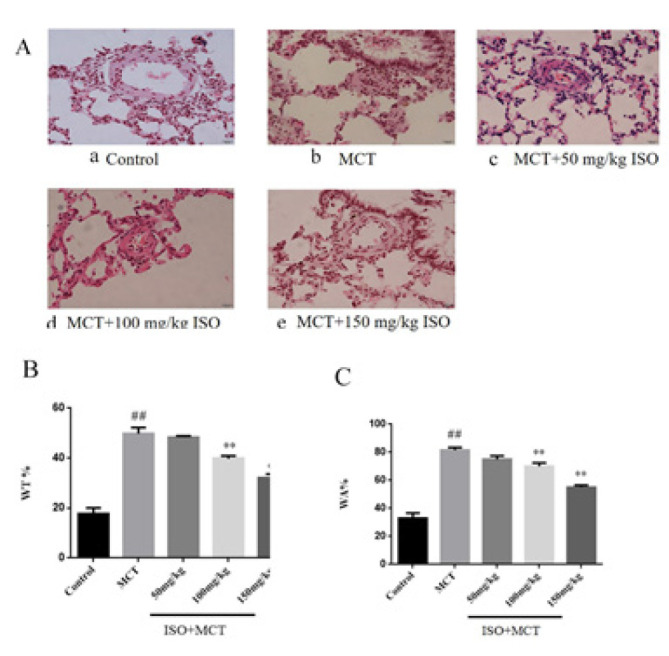
Effect of isorhamnetin on MCT-induced pulmonary vascular remodeling in male SD rats

By observing pathological sections of lung tissue with HE staining, changes in the thickening or thinning of pulmonary blood vessels can be well observed. Compared with the control group, the WT% and WA% in the model group significantly increased, indicating that pulmonary vascular remodeling occurred in the rats with PAH (Figure 4). Compared with the model group, ISO (150 mg/kg) could inhibit WT% and WA% of the model group rats, which indicated that ISO helps improve pulmonary vascular remodeling (Figure 4).

***Effects of*** ***ISO*** ***on SOD activity and MDA content in rat lung tissue***

**Figure 5:**
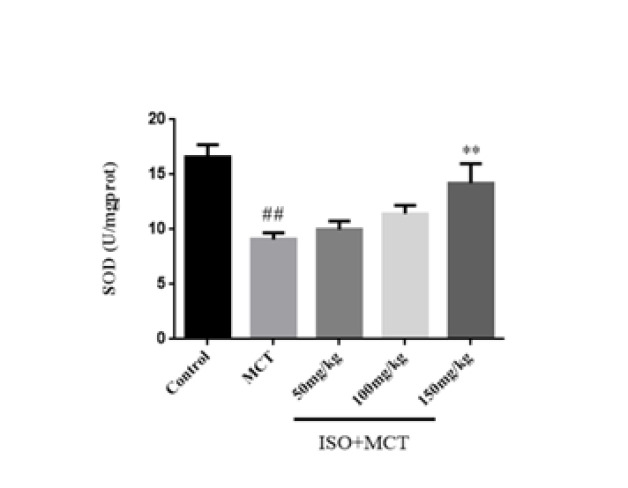
Effect of isorhamnetin on the expression of SOD in male SD rats

**Figure 6:**
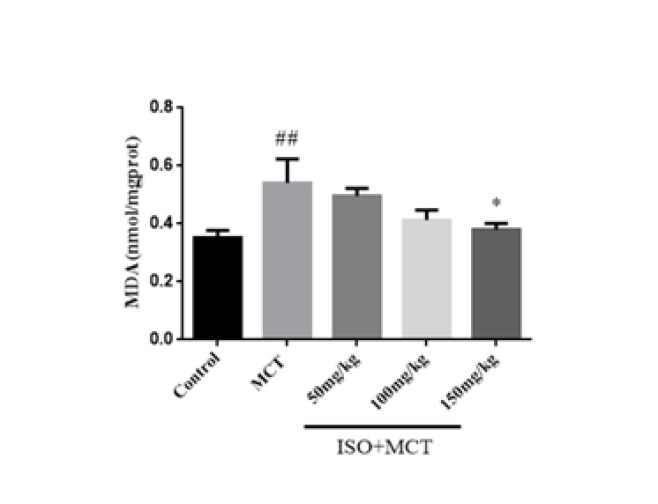
Effect of isorhamnetin on the expression of MDA in male SD rats

The optimal inhibitory concentration of ISO on oxidative stress was studied using an oxidative stress kit and the Elisa method. Both SOD and MDA are the most representative and important indicators of oxidative stress response. SOD and MDA were determined to observe the degree of oxidative stress. Compared with the control group, the SOD activity in the model group was significantly decreased, and the MDA content was significantly increased, which indicated that there was oxidative stress in the PAH rats (Figures 5 and 6). Compared with the model group, ISO (150 mg/kg) could increase SOD activity and decrease MDA content (Figure 6).

***Effect of*** ***ISO*** ***on the content of 5-HT in rat lung tissue***

**Figure 7:**
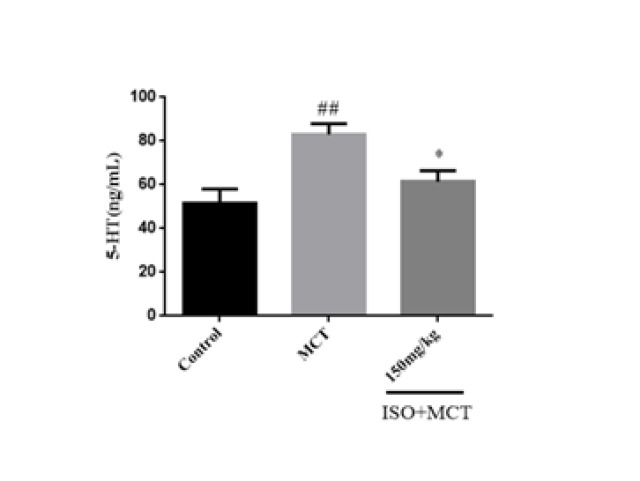
Effect of isorhamnetin on the expression of 5-HT in male SD rats

5-HT is related to regulating the body’s oxidative stress and antioxidant systems and is highly correlated with NADPH. The ability of ISO to resist oxidative stress is explained by measuring 5-HT. The results of the Elisa method showed that compared with the control group, the content of 5-HT in pulmonary tissue homogenate of the model group was significantly increased (Figure 7). Compared with the model group, ISO (150 mg/kg) could reduce the content of 5-HT in lung tissue (Figure 7).

***Effects of*** ***ISO*** ***on protein expression of p-c-src, 5-HTT, NOX1, PCNA and Nrf2 in rat lung tissue***

**Figure 8:**
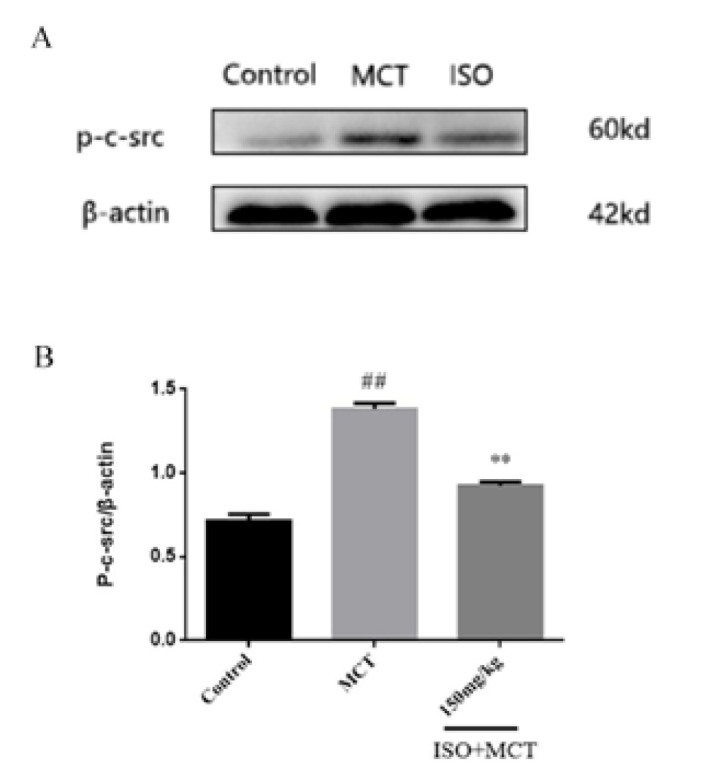
Effect of isorhamnetin on the expression of p-c-src in male SD rats

**Figure 9:**
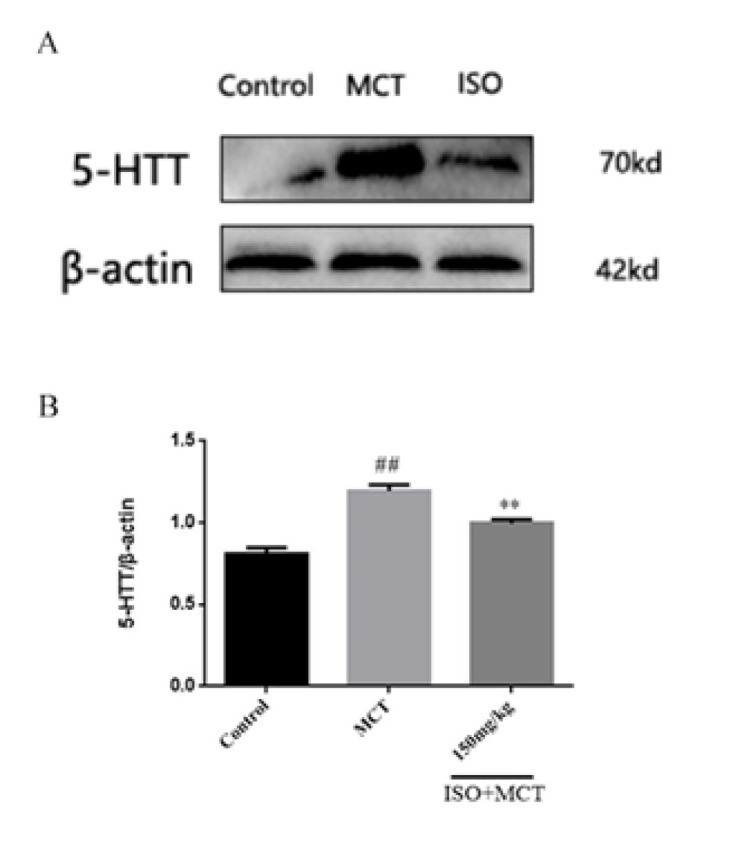
Effect of isorhamnetin on the expression of 5-HTT in male SD rats

**Figure 10:**
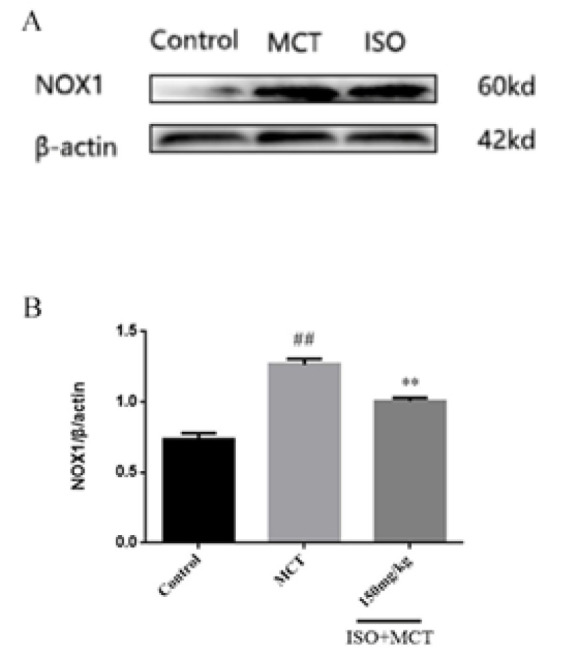
Effect of isorhamnetin on the expression of NOX1 in male SD rats

**Figure 11:**
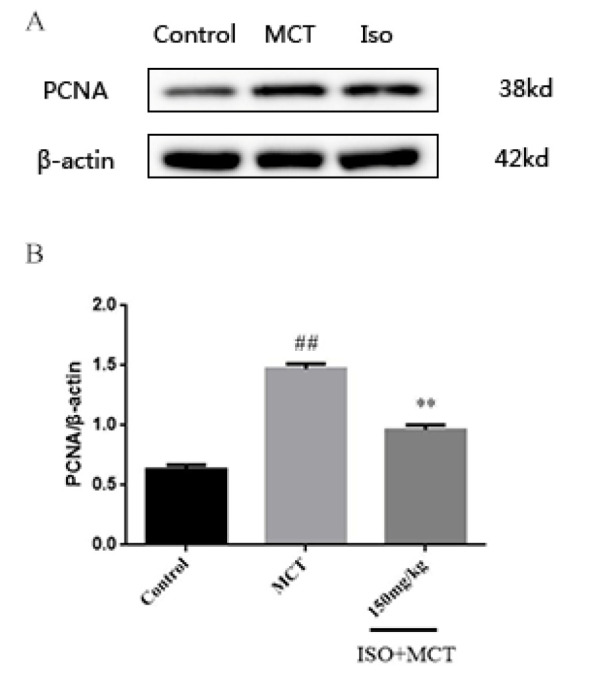
Effect of isorhamnetin on the expression of PCNA in male SD rats

**Figure 12:**
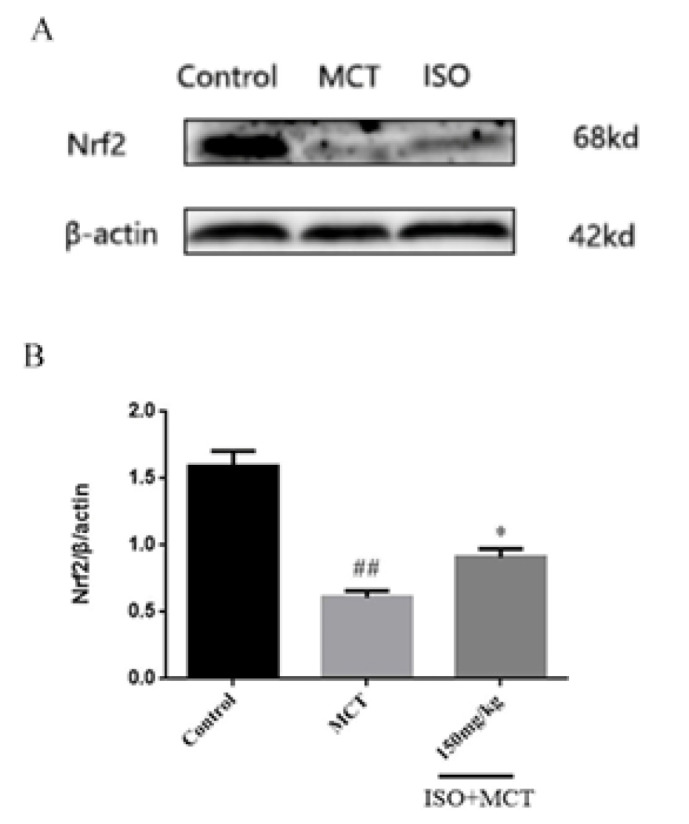
Effect of isorhamnetin on the expression of Nrf2 in male SD rats

Based on the above studies, we found that ISO can counteract MCT-induced oxidation. To further investigate the link between ISO and antioxidant effects, we examined the influence of ISO on the protein expression of 5-HTT, p-c-src, NOX1, PCNA, and Nrf2, which could be related to antioxidant effects. The results of the western-blot method showed that, compared with the control group, the expression of 5-HTT, p-c-src, NOX1, and PCNA (Figures 8, 9, 10, and 11) protein in the model group was significantly increased, while the expression of Nrf2 (Figure 12) protein was significantly decreased. Compared with the model group, ISO (150 mg/kg) could significantly reduce the expression of 5-HTT, p-c-src, NOX1, and PCNA (Figures 8, 9, 10, and 11) protein in the lung tissue of rats, while the expression of Nrf2 (Figure 12) protein was significantly increased *P*<0.05).

Control group; MCT group; ISO+MCT 50 mg/kg group; ISO+MCT 100 mg/kg group; ISO+MCT 150 mg/kg group. Data are expressed as mean±SEM (n=6). #*P<*0.05 vs control group, *P<0.05, **P<0.01 vs MCT group

MCT, monocrotaline; ISO, isorhamnetin; mPAP, mean pulmonary arterial pressure

Control group; MCT group; ISO+MCT 50 mg/kg group; ISO+MCT 100 mg/kg group; ISO+MCT 150 mg/kg group. Data are expressed as mean±SEM (n=6). #*P<*0.05 vs control group, *P<0.05, **P<0.01 vs MCT group

MCT, monocrotaline; ISO, isorhamnetin; RVSP, right ventricular systolic pressure

Control group; MCT group; ISO+MCT 50 mg/kg group; ISO+MCT 100 mg/kg group; ISO+MCT 150 mg/kg group. Data are expressed as mean±SEM (n=6). #*P<*0.05 vs control group, *P<0.05, **P<0.01 vs MCT group

MCT, monocrotaline; ISO, isorhamnetin; RVMI, Right ventricular mass index

Representative photomicrographs of pulmonary small arteries by hematoxylin-eosin (H.E) staining (magnification ×400). (A)(a) Control group; (b) MCT group; ISO+MCT 50 mg/kg group; (d) ISO+MCT 100 mg/kg group; (e) ISO+MCT 150 mg/kg group. (B) Effect of ISO on WT%. (C) Effect of Iso on WA%. Data are expressed as mean±SEM (n=6). #*P<*0.05 vs control group, *P<0.05, **P<0.01 vs MCT group

MCT, monocrotaline; ISO, isorhamnetin; WT%, the ratio of the vascular walls thickness; WA%, the ratio of the vascular wall area

Control group; MCT group; ISO+MCT 50 mg/kg group; ISO+MCT 100 mg/kg group; ISO+MCT 150 mg/kg group. Data are expressed as mean±SEM (n=6). #*P<*0.05 vs control group, *P<0.05, **P<0.01 vs MCT group

MCT, monocrotaline; ISO, isorhamnetin, SOD, Superoxide dismutase

Control group; MCT group; ISO+MCT 50 mg/kg group; ISO+MCT 100 mg/kg group; ISO+MCT 150 mg/kg group. Data are expressed as mean±SEM (n=6). #*P<*0.05 vs control group, *P<0.05, **P<0.01 vs MCT group

MCT, monocrotaline; ISO, isorhamnetin, MDA, malonaldehyde

Control group; MCT group; ISO+MCT 150 mg/kg group. Data are expressed as mean±SEM (n=6). #*P<*0.05 vs control group, *P<0.05, **P<0.01 vs MCT group. MCT, monocrotaline; ISO, isorhamnetin, 5-HT, 5-hydroxytryptamine

(A) Representative western blot band of p-c-src activation in the lung tissues. (B) Effect of isorhamnetin on the protein level of p-c-src in the lung tissue by western blotting. Control group; MCT group; ISO+MCT 150 mg/kg group. Data are expressed as mean±SEM (n=6). #*P<*0.05 vs control group, *P<0.05, **P<0.01 vs MCT group

MCT, monocrotaline; ISO, isorhamnetin, p-c-src, phosphorylation of c-Src tyrosine kinase

(A) Representative western blot band of 5-HTT activation in the lung tissues. (B) Effect of isorhamnetin on the protein level of 5-HTT in the lung tissue by western blotting. Control group; MCT group; ISO+MCT 150 mg/kg group. Data are expressed as mean±SEM (n=6). #*P<*0.05 vs control group, *P<0.05, **P<0.01 vs MCT group

MCT, monocrotaline; ISO, isorhamnetin, 5-HTT, 5-hydroxytryptamine transporter

(A) Representative western blot band of NOX1 activation in the lung tissues. (B) Effect of isorhamnetin on the protein level of NOX1 in the lung tissue by western blotting. Control group; MCT group; ISO+MCT 150 mg/kg group. Data are expressed as mean±SEM (n=6). #*P<*0.05 vs control group, *P<0.05, **P<0.01 vs MCT group

MCT, monocrotaline; ISO, isorhamnetin, NOX1, nicotinamide adenine dinucleotide phosphate (NADPH) oxidase-1

(A) Representative western blot band of PCNA activation in the lung tissues. (B) Effect of isorhamnetin on the protein level of PCNA in the lung tissue by western blotting. Control group; MCT group; ISO+MCT 150 mg/kg group. Data are expressed as mean±SEM (n=6). #*P<*0.05 vs control group, *P<0.05, **P<0.01 vs MCT group

MCT, monocrotaline; ISO, isorhamnetin, PCNA, Proliferating cell nuclear antigen

(A) Representative western blot band of Nrf2 activation in the lung tissues. (B) Effect of isorhamnetin on the protein level of Nrf2 in the lung tissue by western blotting. Control group; MCT group; ISO+MCT 150 mg/kg group. Data are expressed as mean±SEM (n=6). #*P<*0.05 vs control group, *P<0.05, **P<0.01 vs MCT group

MCT, monocrotaline; ISO, isorhamnetin, Nrf2, Nuclear Factor erythroid 2-Related Factor 2

## Discussion

The pathophysiological process of PAH is very complex, and pulmonary vascular remodeling is an important sign. Among all the potential factors of pulmonary vascular remodeling, the proliferation of pulmonary artery smooth muscle cells plays the most important role (14, 15). Previous studies have shown that inflammatory signals, vasoactive substances, oxidative stress, and other signal pathways are jointly involved in the proliferation of smooth muscle cells in the pulmonary artery, ultimately leading to PAH. The main drugs for treating PAH include endothelin receptor antagonists, prostaglandin inhibitors, and phosphodiesterase inhibitors. Still, clinical evidence shows that these drugs can only improve some symptoms of PAH but can not fundamentally reduce mortality (16-18).

Traditional Chinese medicine has a long history of use in China. It has the characteristics of low side effects, low price, and high acceptance by the masses. Therefore, the application of Chinese medicine in the treatment of PAH is receiving more and more attention (19, 20). Seabuckthorn is a deciduous shrub widely distributed in Asia and Europe. Its berries are rich in vitamins, minerals, and other nutrients and rich in flavonoids, amino acids, and other biologically active substances. These biologically active substances have many positive pharmacological activities, including immunomodulatory, anti-aging, antioxidant, and other functions (21, 22). ISO belongs to flavonoids and widely exists in the plant kingdom. ISO has various pharmacological effects such as good anti-oxidation, lowering blood lipid, lowering blood pressure, and expanding coronary arteries, and has a wide range of application prospects in the prevention and treatment of cardiovascular diseases (23, 24).

In this experiment, a rat model of PAH was established by subcutaneous injection of MCT. When male SD rats were injected with MCT, its metabolite MCT-pyrrole reached the lungs through systemic and pulmonary circulation. MCT increases the pressure in the pulmonary artery by injuring endothelial cells and smooth muscle cells. It can lead to pulmonary artery stenosis and cause PAH (25). In this experiment, it was found that 21 days after subcutaneous injection of MCT, the mPAP and RVSP in MCT-induced PAH rats were significantly increased, which indicated the successful establishment of the rat PAH model.

In previous studies, it was found that mPAP and RVSP in hemodynamic indicators in pulmonary hypertension animal models were abnormally elevated. Therefore, this study investigated the effect of ISO on hemodynamic parameters in MCT-induced PAH rats, and the results showed that ISO can inhibit the abnormal increase of mPAP and RVSP in PAH rats, suggesting that ISO can reduce pulmonary arterial pressure. In addition, pulmonary vascular remodeling is also an important pathophysiological feature of PAH, including pulmonary artery wall thickening, muscularization, adventitial fibrosis, and ultimately right heart failure (26). In previous studies, pulmonary vascular remodeling and right ventricular hypertrophy were often evaluated by observing indicators such as WT%, WA%, and RVMI. In this study, the results of HE staining showed that ISO could significantly reduce WT%, WA%, and RVMI in PAH rats, suggesting that ISO has obvious effects on pulmonary vascular remodeling and right ventricular hypertrophy in PAH rats. 

Oxidative stress plays an important role in the formation and development of PAH (27). Studies found that the content of MDA in the lung tissue of MCT-induced PAH rats was significantly increased, and antioxidant factors such as SOD and Nrf2 were significantly decreased, suggesting that there may be extensive oxidative stress in PAH rats. SOD often exerts antioxidant capacity by catalyzing the conversion of superoxide radicals into hydrogen peroxide, and it can be used for the rapid conversion of superoxide radicals to hydrogen peroxide, thereby reducing the level of oxidative stress in the body. MDA is an important product of lipid peroxidation in cell biofilms caused by excess ROS, which can have toxic effects on cells (28, 29). SOD and MDA are often used as important indicators to measure the degree of oxidative stress *in vivo*. Through the detection of oxidative stress kit, it was found that the activity of SOD in the model group was significantly decreased, and the content of MDA was significantly increased. After ISO administration, SOD activity increased, and MDA content decreased in rats, indicating that ISO has an inhibitory effect on oxidative stress. 

Nrf2 is a pivotal factor in the body‘s anti-oxidant system and a key component in maintaining the body‘s redox balance. When the body is subjected to various stimuli and oxidative stress, Nrf2, and related signaling pathways are immediately activated to promote the expression of Recombinant NADH Dehydrogenase, Quinone 1 (NQO1), SODs, and other related antioxidant enzymes, thereby reducing the ROS level in the body(30, 31). It was found by the western-blot method that the expression of Nrf2 in the model group was significantly decreased, compared with that in the control group, and ISO could increase the expression of Nrf2 protein, indicating that ISO can inhibit oxidative stress. 

Pulmonary vascular remodeling is the main pathological feature in the formation of PAH, mainly including pulmonary vascular wall thickening and pulmonary vascular obstruction. Studies have found that pulmonary vascular endothelial cells, pulmonary artery smooth muscle cells, and fibroblasts are all involved in pulmonary vascular remodeling. The proliferation and hypertrophy of pulmonary artery smooth muscle cells are an important cause of pulmonary vascular remodeling and a fundamental cause of PAH. The balance between proliferation and apoptosis of pulmonary artery smooth muscle cells is similar to tumor cells. The continuous proliferation and apoptosis of pulmonary artery smooth muscle cells promote pulmonary vascular remodeling and eventually lead to the formation of PAH (32, 33). PCNA is an important regulatory protein of DNA replication and repair, involved in cell S cycle control and apoptosis, and is an important marker protein for cell proliferation (34). 5-HT, an endogenous vasoactive substance, has been found to play a key role in the development of PAH. It is found that the formation and development of PAH are related to the increase of plasma 5-HT concentration, the up-regulation of the 5-HT transporter, and the enhancement of pulmonary vasoconstriction induced by 5-HT. 5-HT transporter is an NA^+^/Cl^- ^coupled transporter expressed in various tissues, but its expression is higher in the lung. When the body is stimulated by inflammation, hypoxia, and other stimuli, 5-HT is released in large quantities and then combines with 5-HT transporter in large quantities, thereby inducing the proliferation, contraction, and other pathophysiological processes of pulmonary vascular smooth muscle cells (35, 36). In this experiment, it was found by the Elisa method that the 5-HT content in the lung tissue of the model group was significantly higher than that of the control group, while ISO could reduce the 5-HT content. In addition, western-blot found that the protein expression of PCNA and 5-HTT in the model group was significantly increased compared with the control group, while ISO could reduce their protein expression. The above results suggest an abnormal proliferation of pulmonary artery smooth muscle cells induced by 5-HT in PAH rats, and ISO has an inhibitory effect on it.

Studies have shown that NADPH is one of the key enzymes in oxidative stress, and NADPH includes NOX1, NOX2, NOX3, NOX4, NOX5, Dual Oxidase 1, and Dual Oxidase 2. NOX1 is an important member of the NADPH family, and the production of ROS plays a key role in the phenotypic changes of pulmonary artery smooth muscle cells. It has been found that the expression of NOX1 *in vivo* is increased under hypoxia stimulation (37). C-src belongs to a family of non-receptor tyrosine kinases implicated in various cancer disease processes. When c-src is activated, it can promote angiogenesis, proliferation, and migration of invasive cells by activating multiple target proteins (38). Studies have found that c-src is the main non-receptor tyrosine kinase in the vasculature. When c-src is phosphorylated, it can become a proximal regulator of NOX1 activation, affecting the proliferation and apoptosis of pulmonary artery smooth muscle cells. Therefore, the p-c-src/NOX1 signaling pathway may be a potential therapeutic target in the oxidative stress mechanism in PAH. In this experiment, western-blot found that the protein expression of p-c-src and NOX1 in the model group increased significantly compared with that in the control group, and the protein expression of p-c-src and NOX1 decreased significantly compared with that in the model group, indicating that ISO has an inhibitory effect on the p-c-src/NOX1 signal pathway.

## Conclusion

The results show that ISO has certain therapeutic effects on PAH in rats, which is mainly manifested in inhibiting hemodynamic parameters (mPAP and RVSP), relieving pulmonary vascular remodeling (WT% and WA%), reducing right Ventricular hypertrophy (RVMI). In addition, ISO has inhibitory effects on oxidative stress and its related p-c-src/NOX1 signaling pathway, suggesting that ISO has inhibitory effects on oxidative stress and may be related to the inhibition of p-c-src/NOX1 signaling pathway, so that the oxidation and antioxidant systems in the body reach a state of balance. Although further research is needed, this study suggests that ISO may serve as a potential therapeutic agent to provide more diverse options for treating PAH.
